# Real-world safety assessment of burosumab: a pharmacovigilance study utilizing the FDA adverse event reporting system

**DOI:** 10.1186/s13023-026-04267-9

**Published:** 2026-02-28

**Authors:** Tingting Chen, Min Liang

**Affiliations:** The Department of Geriatric Endocrinology and Metabolism, The First Affiliated Hospital of Guangxi Medical University, No. 6 Shuangyong Road, Nanning, Guangxi 530021 China

**Keywords:** Burosumab, Disproportionality analysis, XLH, FAERS, Adverse events

## Abstract

**Objective:**

This study aims to evaluate the adverse events (AEs) associated with burosumab in real-world clinical practice using data from the United States Food and Drug Administration (FDA) Adverse Event Reporting System (FAERS), and to provide evidence for optimizing its clinical safety management.

**Methods:**

Data for this study were retrieved from the FAERS database via OpenVigil 2.1, covering the period from the first quarter of 2018 to the third quarter of 2024. Disproportionality analyses were performed to quantify adverse event signals associated with burosumab, employing four established methods: Reporting Odds Ratio (ROR), Proportional Reporting Ratio (PRR), Multi-Item Gamma Poisson Shrinker (MGPS), and Bayesian Confidence Propagation Neural Network (BCPNN).

**Results:**

A total of 5054 adverse events (AEs) associated with burosumab were identified. Commonly reported AEs included injection site reactions, musculoskeletal pain, restless leg syndrome, skeletal deformities, and corrective orthopedic surgeries necessitated by severe bone deformities. Additionally, several concerning AEs were observed, such as hyperparathyroidism, tertiary hyperparathyroidism, nephrocalcinosis, and dental and periodontal complications.

**Conclusion:**

This study provides an initial evaluation of burosumab’s safety in real-world practice, identifying noteworthy adverse event signals that broaden current understanding and support more informed prescribing decisions for patients with XLH.

**Supplementary Information:**

The online version contains supplementary material available at 10.1186/s13023-026-04267-9.

## Introduction

X-linked hypophosphatemia (XLH) is a rare skeletal disorder caused by loss-of-function mutations in the PHEX gene. These mutations lead to elevated levels of fibroblast growth factor 23 (FGF23), which impair renal phosphate reabsorption and result in hypophosphatemia. Increased FGF23 activity also suppresses renal 1-α-hydroxylase, thereby reducing or inappropriately normalizing circulating levels of 1,25-dihydroxyvitamin D [1,25(OH)_2_D] [1]. Clinically, XLH typically presents in early childhood and is characterized by rickets, growth retardation, limb deformities, chronic pain, and functional impairment.

Burosumab (Crysvita; Kyowa Hakko Kirin Co., Ltd. and Ultragenyx Pharmaceutical Inc.) is a humanized IgG1 monoclonal antibody that binds to and inhibits the activity of excess circulating FGF23 [2]. By inhibiting FGF23 activity, burosumab restores phosphate homeostasis, thereby improving both biochemical parameters and clinical manifestations in patients with XLH [3]. In 2018, burosumab was approved for the treatment of XLH in adults and in children ≥1 year of age in the United States and Canada, as well as in children ≥1 year of age in the European Union [4]. It was later granted approval for the treatment of tumor-induced osteomalacia (TIO) [5].

Several clinical trials have demonstrated the efficacy and safety of burosumab in the treatment of XLH [6–10]. The majority of reported adverse events (AEs) were of mild to moderate severity. The most frequently observed AEs included common pediatric illnesses (e.g., fever, cough, vomiting), symptoms characteristic of XLH (e.g., leg pain), and reactions typically associated with subcutaneous antibody administration (e.g., injection-site irritation). Although burosumab has been available for more than six years, the rarity of XLH has limited the rate at which clinical data can be accumulated. Consequently, existing evidence remains insufficient, underscoring the importance of ongoing pharmacovigilance studies to further evaluate its long-term safety and effectiveness.

The United States Food and Drug Administration (FDA) Adverse Event Reporting System (FAERS) is the largest post-marketing safety surveillance database encompassing all approved drugs and therapeutic biologics [11]. In the present study, we systematically analyzed AEs associated with burosumab by conducting a comprehensive search of the FAERS database. Our objective was to generate a detailed overview of its clinical AE profile, thereby contributing real-world evidence to inform clinical practice and future safety evaluations.

## Methods

### Data source

FAERS (https://fis.fda.gov/extensions/FPD-QDE-FAERS/FPD-QDE-FAERS.html) is a database used for post-marketing surveillance of all FDA-approved drugs and therapeutic biologics. OpenVigil 2.1 (https://openvigil.sourceforge.net/) is a publicly available tool for extracting curated FAERS data [12], including verified and normalized drug names. In this study, OpenVigil 2.1 was employed to retrieve AE data from FAERS spanning the first quarter of 2018 to the third quarter of 2024. Only reports listing “burosumab” or “Crysvita” as the primary suspected (PS) drug were included, while all other reports were excluded. Adverse events were standardized according to preferred terms (PTs), system organ classes (SOCs), and Standardized MedDRA Queries (SMQs) in MedDRA version 27.0, with Microsoft Excel 2021 used for data matching and processing.

### Statistical analysis

A descriptive analysis was performed to summarize the demographic and clinical characteristics of case reports associated with burosumab, including factors such as year of report submission, age, sex, clinical outcome, reporting country, and indication. Each individual safety report was considered a distinct AE report.

Disproportionality analysis (DA) is widely recognized as a key method for detecting drug-related safety signals. In this study, DA was conducted using two-by-two contingency tables (see Supplementary Table S1) and four established algorithms: Reporting Odds Ratio (ROR) [13], Proportional Reporting ratio (PRR) [14], Multi-item Gamma Poisson Shrinker (MGPS) [15], and Bayesian Confidence Propagation Neural Network (BCPNN) [16]. Detailed algorithms and threshold criteria for each method are provided in Supplementary Table S2.

The ROR and PRR methods assess the association between a drug and an AE by calculating the respective ratios and their 95% confidence intervals based on frequency counts. Higher ROR and PRR values indicate stronger signals and a greater association between the target drug and AEs. The BCPNN method evaluates the association between a drug and an AE using the information component (IC), a logarithmic measure of disproportionality. A high IC and its 95% lower confidence limit indicate a statistically significant and strong association between the drug and the AE. The empirical Bayesian geometric mean (EBGM) is a Bayesian method that assesses the strength of the association between a drug and an AE, while adjusting for the overall AE reporting rate across all drugs and reducing sensitivity to random variation. AEs that met all of the four method thresholds were considered potential signals. All analyses were performed using Microsoft Excel 2021.

## Results

### Descriptive results

A total of 7,480,032 adverse event reports were retrieved, of which 5,054 (0.0676%) identified burosumab as the primary suspected drug, spanning from the first quarter of 2018 to the third quarter of 2024. Clinical characteristics of these reports were summarized in Table 1. For the majority of reported AEs, information on sex, age, and serious outcomes was unavailable. The United States accounted for the largest proportion of reports (93.45%), followed by the United Kingdom (2.24%). Among serious outcomes, hospitalization was the most frequently reported (6.33%), alongside 7 life-threatening cases (0.14%) and 29 deaths (0.57%). The most commonly reported indication for burosumab was hereditary hypophosphatemic rickets (78.35%).

**Table 1:** Clinical characteristics of reports associated with burosumab (2018 Q1-2024 Q3)

Characteristics	Number of cases	Proportion of cases (%)
Number of adverse events	5054	
Year of report		
2018	75	1.48
2019	576	11.40
2020	61	1.21
2021	1113	22.02
2022	1717	33.97
2023	924	18.28
2024	588	11.63
Sex		
Female	14	0.28
Male	9	0.18
Unknown	5031	99.54
Age		
＜18	8	0.16
≥18	7	0.14
Unknown	5039	99.70
Reported countries		
United States	4723	93.45
United Kindom	113	2.24
Germany	49	0.97
France	41	0.81
Other	128	2.53
Serious outcome		
Death	29	0.57
Life threatening	7	0.14
Hospitalization	320	6.33
Disability	10	0.20
Other	606	11.99
Unknown	4082	80.77
Indication (Top7)		
Hereditary hypophosphataemic rickets	3960	78.35
Product used for unknown indication	741	14.66
Hypophosphataemic osteomalacia	96	1.90
Hypophosphataemia	93	1.84
Phosphorus metabolism disorder	87	1.72
Osteomalacia	39	0.77
Rickets	10	0.20

### Adverse events at the SOC levels

Adverse events at the SOC level are summarized in Table 2. A total of 15 SOCs were associated with burosumab. Among these, injury, poisoning, and procedural complications (*n* = 1282) accounted for the highest number of case reports. The top 10 SOCs further included investigations (*n* = 755), musculoskeletal and connective tissue disorders (*n* = 749), general disorders and administration site conditions (*n* = 622), infections and infestations (*n* = 310), surgical and medical procedures (*n* = 208), nervous system disorders (*n* = 165), gastrointestinal disorders (*n* = 77), renal and urinary disorders (*n* = 43), and metabolism and nutrition disorders (*n* = 31).

**Table 2:** Burosumab adverse events were ranked in descending order by case reports at the SOC level in FAERS database

SOC	Case reports	ROR (95% Cl)	PRR (χ^2^)	EBGM (EBGM05)	IC (IC025)
Injury, poisoning and procedural complications	1282	8.83 (8.41–9.27)	6.13 (5813.47)	6.11 (5.85)	2.61 (2.49)
Investigations	755	53.89 (50.87–57.08)	43.14 (30571.46)	42.25 (40.09)	5.40 (5.10)
Musculoskeletal and connective tissue disorders	749	5.54 (5.23–5.86)	4.62 (2218.37)	4.61 (4.38)	2.21 (2.08)
General disorders and administration site conditions	622	4.75 (4.47–5.05)	4.12 (1529.65)	4.11 (3.89)	2.04 (1.92)
Infections and infestations	310	5.01 (4.61–5.44)	4.67 (909.36)	4.67 (4.32)	2.22 (2.04)
Surgical and medical procedures	208	6.02 (5.44–6.65)	5.74 (818.98)	5.72 (5.22)	2.52 (2.28)
Nervous system disorders	165	54.70 (48.86–61.24)	52.31 (8101.78)	51.01 (46.01)	5.67 (5.07)
Gastrointestinal disorders	77	11.39 (9.69–13.39)	11.17 (710.72)	11.12 (9.59)	3.47 (2.96)
Renal and urinary disorders	43	5.98 (4.82–7.42)	5.92 (175.85)	5.91 (4.85)	2.56 (2.07)
Metabolism and nutrition disorders	31	7.62 (5.92–9.82)	7.57 (176.23)	7.54 (5.98)	2.92 (2.26)
Social circumstances	22	6.36 (4.71–8.59)	6.33 (98.50)	6.31 (4.80)	2.66 (1.97)
Congenital, familial and genetic disorders	20	83.28 (60.46–114.72)	82.84 (1553.23)	79.61 (59.39)	6.31 (4.58)
Psychiatric disorders	16	5.94 (4.18–8.43)	5.91 (65.19)	5.90 (4.28)	2.56 (1.80)
Endocrine disorders	13	58.35 (39.33–86.58)	58.15 (709.76)	56.55 (39.42)	5.82 (3.92)
Product issues	9	4.55 (2.85–7.27)	4.55 (24.84)	4.54 (2.96)	2.18 (1.37)

Abbreviations: SOC, System Organ Class; CI, confidence interval; ROR, reporting odds ratio; PRR, proportional reporting ratio; EBGM, empirical Bayesian geometric mean; EBGM05, the lower limit of the 95% CI of EBGM; IC, information component; IC025, the lower limit of the 95% CI of the IC

### Adverse events at the PT levels

The top 30 adverse events associated with burosumab at the PT level, ranked by the IC025 value, are presented in Table 3. The highest-ranking PT-level AEs included blood phosphorus decreased, blood 25-hydroxycholecalciferol decreased, knee deformity, blood phosphorus increased, blood phosphorus abnormal, restless legs syndrome, osteotomy, blood parathyroid hormone increased, and nephrocalcinosis. All AEs with positive safety signals are provided in Supplementary Table S3.

**Table 3:** The top 30 adverse events of burosumab at the PT level ranked in descending order by IC025 value in FAERS database

PT	Case reports	ROR (95% Cl)	PRR (χ^2^)	EBGM (EBGM05)	IC (IC025)
Blood phosphorus decreased	429	1348.43 (1233.94–1473.55)	1192.79 (320826.02)	749.36 (690.94)	9.55 (8.74)
Blood 25-hydroxycholecalciferol decreased	22	450.00 (323.26–626.42)	447.34 (8016.21)	366.18 (270.58)	8.52 (6.12)
Knee deformity	44	207.00 (165.67–258.64)	204.56 (8091.03)	185.78 (151.54)	7.54 (6.03)
Blood phosphorus increased	43	162.16 (129.74–202.68)	160.30 (6305.40)	148.55 (121.13)	7.21 (5.77)
Blood phosphorus abnormal	20	308.99 (220.61–432.76)	307.33 (5297.94)	266.76 (196.01)	8.06 (5.75)
Restless legs syndrome	152	68.42 (60.82–76.97)	65.66 (9379.00)	63.62 (57.12)	5.99 (5.33)
Osteotomy	10	480.58 (293.49–786.93)	479.29 (3855.06)	387.31 (246.69)	8.60 (5.25)
Blood parathyroid hormone increased	34	106.92 (83.48–136.93)	105.95 (3358.13)	100.70 (80.31)	6.65 (5.20)
Nephrocalcinosis	23	136.22 (100.66–184.32)	135.38 (2874.68)	126.91 (96.24)	6.99 (5.16)
Fibroblast growth factor 23 increased	8	896.60 (494.37–1626.10)	894.67 (4944.05)	619.70 (359.48)	9.28 (5.11)
Tooth abscess	75	64.97 (55.04–76.69)	63.68 (4486.47)	61.75 (53.06)	5.95 (5.04)
Vitamin D decreased	69	65.78 (55.34–78.18)	64.57 (4185.66)	62.60 (53.45)	5.97 (5.02)
Craniosynostosis	12	141.72 (93.24–215.41)	141.26 (1561.70)	132.07 (90.04)	7.05 (4.63)
Hyperparathyroidism tertiary	5	559.92 (275.80–1136.72)	559.17 (2180.24)	437.83 (229.09)	8.77 (4.32)
Restless arm syndrome	6	159.18 (87.87–288.34)	158.92 (872.68)	147.37 (85.58)	7.20 (3.98)
Limb operation	26	33.08 (25.06–43.67)	32.86 (790.30)	32.34 (25.09)	5.02 (3.80)
Growth accelerated	5	167.98 (87.51–322.43)	167.75 (765.04)	154.92 (85.32)	7.28 (3.79)
Exposure to SARS-CoV-2	16	30.95 (21.74–44.07)	30.82 (454.76)	30.37 (21.98)	4.92 (3.46)
Vitamin D abnormal	5	97.85 (51.52–185.85)	97.72 (456.49)	93.24 (51.85)	6.54 (3.44)
Arnold-Chiari malformation	8	51.23 (31.02–84.62)	51.12 (383.43)	49.88 (31.52)	5.64 (3.42)
Quarantine	5	92.46 (48.72–175.48)	92.34 (431.95)	88.33 (49.16)	6.46 (3.41)
Contraindicated product administered	93	15.27 (13.17–17.69)	14.91 (1200.02)	14.81 (12.94)	3.89 (3.35)
Limb deformity	9	41.65 (25.98–66.78)	41.55 (349.01)	40.73 (26.45)	5.35 (3.34)
Dental caries	24	20.49 (15.36–27.33)	20.36 (437.51)	20.17 (15.49)	4.33 (3.25)
Bone pain	103	13.24 (11.51–15.23)	12.90 (1125.83)	12.82 (11.28)	3.68 (3.20)
Blood alkaline phosphatase increased	37	16.08 (12.75–20.29)	15.93 (514.06)	15.81 (12.79)	3.98 (3.16)
Hyperparathyroidism	8	37.36 (22.66–61.59)	37.28 (277.32)	36.62 (23.18)	5.19 (3.15)
Growth disorder	5	62.99 (33.34–119.01)	62.91 (295.39)	61.03 (34.10)	5.93 (3.14)
Endodontic procedure	9	32.08 (20.03–51.38)	32.01 (266.15)	31.52 (20.49)	4.98 (3.11)
Vitamin D increased	5	57.92 (30.68–109.35)	57.85 (271.52)	56.26 (31.46)	5.81 (3.08)

Abbreviations: PT, preferred term; CI, confidence interval; ROR, reporting odds ratio; PRR, proportional reporting ratio; χ2, chi-squared; IC, information component; IC025, the lower limit of 95% CI of the IC; EBGM, empirical Bayesian geometric mean; EBGM05, the lower limit of 95% CI of EBGM

### Adverse events at the SMQ levels

**Fig. 1:**
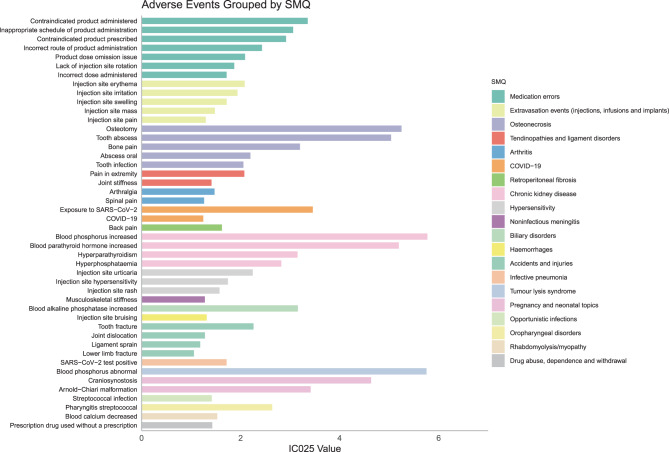
PT-level adverse events associated with burosumab, grouped by SMQ and ranked by IC025 values in descending order

Adverse events at the SMQ levels are demonstrated in Table 4. A total of 20 SMQs were identified for burosumab. The most frequently reported SMQs included medication errors; extravasation events (injections, infusions, and implants); osteonecrosis; tendinopathies and ligament disorders; and arthritis. Furthermore, burosumab-related AEs at the PT level, grouped by SMQ, are illustrated in Fig. 1. The top five PTs and their corresponding SMQs, ranked by IC025 values, were as follows: blood phosphorus increased in chronic kidney disease, blood phosphorus abnormal in tumor lysis syndrome, osteotomy in osteonecrosis, blood parathyroid hormone increased in chronic kidney disease, and tooth abscess in osteonecrosis.

**Table 4:** Burosumab adverse events were ranked in descending order by IC025 value at the SMQ level in FAERS database

SMQ	Case reports	ROR (95% Cl)	PRR (χ^2^)	EBGM (EBGM05)	IC (IC025)
Medication errors	1223	8.62 (8.20–9.05)	6.11 (5505.70)	6.09 (5.83)	2.61 (2.48)
Extravasation events (injections, infusions and implants)	298	3.85 (3.53–4.19)	3.62 (576.32)	3.61 (3.34)	1.85 (1.70)
Osteonecrosis	207	19.35 (17.50–21.40)	18.33 (3371.48)	18.17 (16.58)	4.18 (3.78)
Tendinopathies and ligament disorders	204	5.11 (4.62–5.65)	4.89 (635.95)	4.88 (4.45)	2.29 (2.07)
Arthritis	203	3.26 (2.95–3.61)	3.14 (300.39)	3.13 (2.86)	1.65 (1.49)
COVID-19	154	3.02 (2.69–3.39)	2.93 (198.90)	2.93 (2.64)	1.55 (1.38)
Retroperitoneal fibrosis	114	3.71 (3.24–4.23)	3.62 (217.93)	3.62 (3.20)	1.86 (1.62)
Chronic kidney disease	92	89.85 (77.26–104.48)	87.65 (7553.95)	84.03 (73.20)	6.39 (5.50)
Hypersensitivity	54	6.19 (5.11–7.51)	6.12 (231.11)	6.10 (5.12)	2.61 (2.15)
Noninfectious meningitis	39	3.06 (2.44–3.83)	3.04 (53.40)	3.03 (2.47)	1.60 (1.28)
Biliary disorders	37	16.08 (12.75–20.29)	15.93 (514.06)	15.81 (12.79)	3.98 (3.16)
Haemorrhages	37	3.18 (2.52–4.01)	3.16 (54.64)	3.15 (2.55)	1.66 (1.31)
Accidents and injuries	35	5.14 (4.05–6.53)	5.11 (115.48)	5.10 (4.10)	2.35 (1.85)
Infective pneumonia	21	5.07 (3.73–6.90)	5.05 (68.14)	5.04 (3.81)	2.33 (1.72)
Tumour lysis syndrome	20	308.99 (220.61–432.76)	307.33 (5297.94)	266.76 (196.01)	8.06 (5.75)
Pregnancy and neonatal topics	20	83.28 (60.46–114.72)	82.84 (1553.23)	79.61 (59.39)	6.31 (4.58)
Oropharyngeal disorders	20	12.39 (9.05–16.98)	12.33 (207.06)	12.26 (9.19)	3.62 (2.64)
Opportunistic infections	13	4.97 (3.36–7.33)	4.95 (40.94)	4.94 (3.46)	2.31 (1.56)
Rhabdomyolysis/myopathy	8	5.73 (3.49–9.41)	5.72 (31.06)	5.70 (3.62)	2.51 (1.53)
Drug abuse, dependence and withdrawal	8	5.10 (3.10–8.37)	5.09 (26.21)	5.08 (3.22)	2.34 (1.43)
NA	1515				

Abbreviations: SMQ, Standardised MedDRA Query; CI, confidence interval; ROR, reporting odds ratio; PRR, proportional reporting ratio; χ2, chi-squared; IC, information component; IC025, the lower limit of 95% CI of the IC; EBGM, empirical Bayesian geometric mean; EBGM05, the lower limit of 95% CI of EBGM; NA, not applicable

## Discussion

This study analyzed FAERS data on adverse events associated with burosumab since its approval in 2018 to provide comprehensive safety information for healthcare professionals and patients. To more accurately assess AEs more likely attributable to burosumab, reports potentially related to non-pharmacologic factors (e.g., product issues and medication errors) and certain pandemic-related conditions (e.g., COVID-19) were excluded from the subsequent detailed discussion.

In the disproportionality analysis, several AEs with high signal strength—such as hypophosphatemia, elevated FGF23 levels, increased alkaline phosphatase (ALP) levels, and reduced 1,25[OH]_2_D levels—were identified. These events represent hallmarks of XLH and are directly involved in its pathogenesis; therefore, they are more appropriately interpreted as disease-related complications rather than drug-induced AEs [17]. Previous studies have reported that the most common AEs associated with burosumab include injection site reactions, musculoskeletal pain, restless leg syndrome, skeletal deformities, and corrective orthopedic surgeries for severe bone deformities [7, 18, 19]. Consistent with previous findings, these events were also observed in our analysis, further supporting the reliability of our analytical methodology.

Our study identified both hyperparathyroidism and tertiary hyperparathyroidism as adverse events associated with burosumab treatment. Hyperparathyroidism is a common complication in patients with XLH [20, 21]. Previous studies reported that 4 of 37 pediatric patients developed secondary hyperparathyroidism, while none developed tertiary hyperparathyroidism [22]. Although tertiary hyperparathyroidism is extremely rare in pediatric patients, two cases were observed during burosumab treatment, raising concern about a possible association [23]. These observations warrant further investigation to clarify the relationship between burosumab and hyperparathyroidism. Moreover, close monitoring of parathyroid function is recommended for patients receiving burosumab.

Nephrocalcinosis (NC) was identified as an adverse event with high signal strength in our study, suggesting a potential renal safety signal in patients with XLH. Calcium deposition within the renal tubules may lead to kidney damage over time [24]. Conventional therapy, including phosphate supplements and active vitamin D, has been associated with an increased risk of NC [25]. A large longitudinal study suggested NC is more likely a complication of conventional therapy rather than an intrinsic feature of XLH [26]. Supporting this notion, a retrospective single-center study reported a significant reduction in urinary phosphate levels in children with XLH who transitioned from conventional therapy to burosumab [27]. Several studies have indicated that burosumab treatment does not worsen pre-existing NC or induce new cases [7, 28, 29]. However, other reports have presented conflicting findings regarding NC progression during burosumab therapy [9, 30]. In a single-center pediatric cohort, one patient developed NC during burosumab treatment, although the underlying pathophysiology remains unclear [31]. Taken together, these findings highlight the need for further investigation to clarify the potential relationship between burosumab therapy and NC development.

Data on the effects of burosumab treatment on dental and periodontal outcomes remain limited. Patients with XLH commonly experience dental and periodontal complications, including spontaneous periapical abscesses, defective dentin mineralization, and early tooth loss [32]. Insogna et al. reported a higher incidence of dental abscesses in adult patients treated with burosumab over a 24-week period [33]. In pediatric patients aged 1–12 years, Ward et al. found that those receiving burosumab were less likely to develop dental abscesses at younger ages (<5 years) compared with conventional therapy, but more likely to develop abscesses at older ages (5–12 years) [34]. Conversely, a recent study indicated that adults with XLH treated with burosumab experienced fewer endodontic infections during a monitoring period of at least six months [35]. The observed heterogeneity in dental outcomes may be attributable to differences in patient characteristics, study design, duration of follow-up, or potential effects of prior conventional therapy. Although preclinical studies in mouse models suggest that burosumab may improve dentoalveolar tissues and potentially provide clinical benefits for dental abnormalities [36], larger clinical studies with extended follow-up are required to clarify the long-term impact of burosumab on dental health.

Interestingly, our analysis identified 9 reported cases of attention deficit hyperactivity disorder (ADHD). Although no prior studies have established a direct association between ADHD and either XLH or burosumab treatment, this observation may warrant attention to neurobehavioral and psychological health in patients receiving burosumab.

This study has several methodological strengths. By leveraging the FAERS database, it assesses adverse events associated with burosumab in real-world settings, enabling the early detection of potential post-marketing safety signals and providing valuable guidance for clinical practice. Furthermore, the application of four complementary analytical methods enhances the comprehensiveness and stability of signal detection, allowing for a more thorough evaluation of burosumab’s adverse event profile in the target population.

This study has several limitations. First, the FAERS database, as a spontaneous reporting system, is inherently subject to underreporting, overreporting of unrelated events, and incomplete background information. The quality and accuracy of these reports are influenced by the expertise of the individuals submitting them [37]. Second, the lack of detailed clinical and medication information restricts the ability to adequately adjust for potential confounders, including comorbidities, concomitant therapies, and dosage, thereby limiting the depth and interpretability of the analysis. For instance, although sexual dimorphism in the severity of XLH has been documented [38], the absence of sex-specific data precludes exploration of sex-related differences in burosumab-associated adverse events. Third, associations observed between drugs and adverse events in FAERS reflect statistical correlations rather than causal relationships and thus require confirmation through more rigorous approaches, such as clinical trials and epidemiological studies. Despite these limitations, pharmacovigilance analyses based on FAERS offer valuable access to large-scale real-world data and play an important role in the early detection of potential drug safety signals. The findings of this study may offer supportive evidence for the continued evaluation of burosumab safety in clinical practice.

## Conclusions

This study provides an initial evaluation of the safety profile of burosumab in real-world clinical practice, identifying several potential new adverse event signals. These findings contribute to the evolving understanding of burosumab-associated risks and may assist clinicians in making more informed clinical decisions for patients with XLH. Nevertheless, further studies are warranted to validate these observations and to comprehensively assess the long-term safety and tolerability of burosumab.

## Electronic supplementary material

Below is the link to the electronic supplementary material.

Supplementary Material 1

## Data Availability

The data that support the findings of this study are available upon from the authors. The raw data can be obtained from the FAERS database at the following link: https://fis.fda.gov/extensions/FPD-QDE-FAERS/FPD-QDE-FAERS.html.
