# Supplementary material for: Real-world safety assessment of burosumab: a pharmacovigilance study utilizing the FDA adverse event reporting system
Source: Orphanet J Rare Dis. 2026 Feb 28;21:131. doi: 10.1186/s13023-026-04267-9 (PMC13059399; doi:10.1186/s13023-026-04267-9)
Supplement: Supplementary file 1 — Supplementary Material 1 [file 13023_2026_4267_MOESM1_ESM.docx]

**Supplementary Table S1.** Two-by-two contingency table for disproportionality analyses.

|  | Target AEs | Other AEs | Total |
| --- | --- | --- | --- |
| Burosumab | a | b | a+b |
| Other drugs | c | d | c+d |
| Total | a+c | b+d | a+b+c+d |

AEs, adverse events; a, number of reports containing both the target drug and target adverse drug reaction; b, number of reports containing other adverse drug reaction of the target drug; c, number of reports containing the target adverse drug reaction of other drugs; d, number of reports containing other drugs and other adverse drug reactions.

**Supplementary Table S2.** Four major algorithms used for signal detection.

| Algorithms | Equation | Criteria |
| --- | --- | --- |
| ROR | ROR=ad/b/c | lower limit of 95% CI>1, N≥3 |
|  | 95%CI=e^ln(ROR)±1.96(1/a+1/b+1/c+1/d)^0.5^ |  |
| PRR | PRR=a(c+d)/c/(a+b) | PRR≥2, χ^2^≥4, N≥3 |
|  | χ^2^=[(ad-bc)^2](a+b+c+d)/[(a+b)(c+d)(a+c)(b+d)] |  |
| BCPNN | IC=log_2_a(a+b+c+d)(a+c)(a+b) | IC025>0 |
|  | 95%CI= E(IC) ± 2V(IC)^0.5 |  |
| MGPS | EBGM=a(a+b+c+d)/(a+c)/(a+b) | EBGM05>2 |
|  | 95%CI=e^ln(EBGM)±1.96(1/a+1/b+1/c+1/d)^0.5^ |  |

a, number of reports containing both the target drug and target adverse drug reaction; b, number of reports containing other adverse drug reaction of the target drug; c, number of reports containing the target adverse drug reaction of other drugs; d, number of reports containing other drugs and other adverse drug reactions. 95%CI, 95% confidence interval; N, the number of reports; χ2, chi-squared; IC, information component; IC025, the lower limit of 95% CI of the IC; E(IC), the IC expectations; V(IC), the variance of IC; EBGM, empirical Bayesian geometric mean; EBGM05, the lower limit of 95% CI of EBGM.

**Supplementary Table S3.** Burosumab adverse events were ranked in descending order by case reports at the PT level in FAERS database.

| **PT** | **Case reports** | **ROR**  **(95% Cl)** | **PRR (χ^2^)** | **EBGM (EBGM05)** | **IC (IC025)** |
| --- | --- | --- | --- | --- | --- |
| Blood phosphorus decreased | 429 | 1348.43 (1233.94-1473.55) | 1192.79 (320826.02) | 749.36 (690.94) | 9.55 (8.74) |
| Blood 25-hydroxycholecalciferol decreased | 22 | 450.00 (323.26-626.42) | 447.34 (8016.21) | 366.18 (270.58) | 8.52 (6.12) |
| Knee deformity | 44 | 207.00 (165.67-258.64) | 204.56 (8091.03) | 185.78 (151.54) | 7.54 (6.03) |
| Blood phosphorus increased | 43 | 162.16 (129.74-202.68) | 160.30 (6305.40) | 148.55 (121.13) | 7.21 (5.77) |
| Blood phosphorus abnormal | 20 | 308.99 (220.61-432.76) | 307.33 (5297.94) | 266.76 (196.01) | 8.06 (5.75) |
| Restless legs syndrome | 152 | 68.42 (60.82-76.97) | 65.66 (9379.00) | 63.62 (57.12) | 5.99 (5.33) |
| Osteotomy | 10 | 480.58 (293.49-786.93) | 479.29 (3855.06) | 387.31 (246.69) | 8.60 (5.25) |
| Blood parathyroid hormone increased | 34 | 106.92 (83.48-136.93) | 105.95 (3358.13) | 100.70 (80.31) | 6.65 (5.20) |
| Nephrocalcinosis | 23 | 136.22 (100.66-184.32) | 135.38 (2874.68) | 126.91 (96.24) | 6.99 (5.16) |
| Fibroblast growth factor 23 increased | 8 | 896.60 (494.37-1626.10) | 894.67 (4944.05) | 619.70 (359.48) | 9.28 (5.11) |
| Tooth abscess | 75 | 64.97 (55.04-76.69) | 63.68 (4486.47) | 61.75 (53.06) | 5.95 (5.04) |
| Vitamin D decreased | 69 | 65.78 (55.34-78.18) | 64.57 (4185.66) | 62.60 (53.45) | 5.97 (5.02) |
| Craniosynostosis | 12 | 141.72 (93.24-215.41) | 141.26 (1561.70) | 132.07 (90.04) | 7.05 (4.63) |
| Hyperparathyroidism tertiary | 5 | 559.92 (275.80-1136.72) | 559.17 (2180.24) | 437.83 (229.09) | 8.77 (4.32) |
| Restless arm syndrome | 6 | 159.18 (87.87-288.34) | 158.92 (872.68) | 147.37 (85.58) | 7.20 (3.98) |
| Limb operation | 26 | 33.08 (25.06-43.67) | 32.86 (790.30) | 32.34 (25.09) | 5.02 (3.80) |
| Growth accelerated | 5 | 167.98 (87.51-322.43) | 167.75 (765.04) | 154.92 (85.32) | 7.28 (3.79) |
| Exposure to SARS-CoV-2 | 16 | 30.95 (21.74-44.07) | 30.82 (454.76) | 30.37 (21.98) | 4.92 (3.46) |
| Vitamin D abnormal | 5 | 97.85 (51.52-185.85) | 97.72 (456.49) | 93.24 (51.85) | 6.54 (3.44) |
| Arnold-Chiari malformation | 8 | 51.23 (31.02-84.62) | 51.12 (383.43) | 49.88 (31.52) | 5.64 (3.42) |
| Quarantine | 5 | 92.46 (48.72-175.48) | 92.34 (431.95) | 88.33 (49.16) | 6.46 (3.41) |
| Contraindicated product administered | 93 | 15.27 (13.17-17.69) | 14.91 (1200.02) | 14.81 (12.94) | 3.89 (3.35) |
| Limb deformity | 9 | 41.65 (25.98-66.78) | 41.55 (349.01) | 40.73 (26.45) | 5.35 (3.34) |
| Dental caries | 24 | 20.49 (15.36-27.33) | 20.36 (437.51) | 20.17 (15.49) | 4.33 (3.25) |
| Bone pain | 103 | 13.24 (11.51-15.23) | 12.90 (1125.83) | 12.82 (11.28) | 3.68 (3.20) |
| Blood alkaline phosphatase increased | 37 | 16.08 (12.75-20.29) | 15.93 (514.06) | 15.81 (12.79) | 3.98 (3.16) |
| Hyperparathyroidism | 8 | 37.36 (22.66-61.59) | 37.28 (277.32) | 36.62 (23.18) | 5.19 (3.15) |
| Growth disorder | 5 | 62.99 (33.34-119.01) | 62.91 (295.39) | 61.03 (34.10) | 5.93 (3.14) |
| Endodontic procedure | 9 | 32.08 (20.03-51.38) | 32.01 (266.15) | 31.52 (20.49) | 4.98 (3.11) |
| Vitamin D increased | 5 | 57.92 (30.68-109.35) | 57.85 (271.52) | 56.26 (31.46) | 5.81 (3.08) |
| Weight abnormal | 12 | 24.85 (16.54-37.35) | 24.78 (270.50) | 24.49 (16.87) | 4.61 (3.07) |
| Inappropriate schedule of product administration | 438 | 10.98 (10.23-11.80) | 9.81 (3488.82) | 9.76 (9.15) | 3.29 (3.06) |
| Medical device removal | 6 | 41.01 (23.01-73.08) | 40.94 (229.15) | 40.15 (23.67) | 5.33 (2.99) |
| Contraindicated product prescribed | 11 | 22.36 (14.62-34.21) | 22.30 (221.36) | 22.07 (14.96) | 4.46 (2.92) |
| Hyperphosphataemia | 7 | 28.52 (16.73-48.62) | 28.47 (182.94) | 28.08 (17.24) | 4.81 (2.82) |
| Emergency care | 13 | 18.13 (12.27-26.80) | 18.07 (207.84) | 17.92 (12.54) | 4.16 (2.82) |
| Toothache | 28 | 12.26 (9.40-16.01) | 12.18 (285.76) | 12.11 (9.49) | 3.60 (2.76) |
| Injection site pruritus | 79 | 9.63 (8.21-11.30) | 9.45 (595.18) | 9.41 (8.13) | 3.23 (2.76) |
| Blood calcium increased | 15 | 15.17 (10.55-21.82) | 15.11 (196.26) | 15.01 (10.76) | 3.91 (2.72) |
| Growth retardation | 8 | 21.32 (12.95-35.08) | 21.27 (152.96) | 21.06 (13.35) | 4.40 (2.67) |
| Pharyngitis streptococcal | 20 | 12.39 (9.05-16.98) | 12.33 (207.06) | 12.26 (9.19) | 3.62 (2.64) |
| Knee operation | 19 | 11.16 (8.08-15.42) | 11.11 (173.96) | 11.06 (8.23) | 3.47 (2.51) |
| Incorrect route of product administration | 38 | 8.43 (6.71-10.60) | 8.35 (245.24) | 8.32 (6.75) | 3.06 (2.43) |
| Ear infection | 32 | 7.64 (5.96-9.80) | 7.59 (182.48) | 7.56 (6.02) | 2.92 (2.28) |
| Tooth fracture | 14 | 9.87 (6.78-14.38) | 9.84 (110.69) | 9.80 (6.95) | 3.29 (2.26) |
| Injection site urticaria | 29 | 7.63 (5.87-9.91) | 7.58 (165.07) | 7.55 (5.94) | 2.92 (2.25) |
| Tooth extraction | 14 | 9.27 (6.37-13.50) | 9.24 (102.45) | 9.20 (6.53) | 3.20 (2.20) |
| Abscess oral | 4 | 22.02 (10.89-44.53) | 22.00 (79.32) | 21.77 (11.43) | 4.44 (2.20) |
| Hyperaesthesia teeth | 5 | 16.69 (8.89-31.30) | 16.66 (73.02) | 16.54 (9.30) | 4.05 (2.16) |
| Product dose omission issue | 467 | 5.27 (4.92-5.65) | 4.74 (1410.16) | 4.73 (4.44) | 2.24 (2.09) |
| Injection site erythema | 79 | 5.54 (4.73-6.50) | 5.45 (287.17) | 5.44 (4.70) | 2.44 (2.08) |
| Pain in extremity | 190 | 5.16 (4.65-5.73) | 4.95 (603.39) | 4.94 (4.49) | 2.30 (2.08) |
| Injection site reaction | 53 | 5.76 (4.74-6.99) | 5.69 (204.73) | 5.68 (4.75) | 2.50 (2.06) |
| Tooth infection | 15 | 7.81 (5.43-11.23) | 7.78 (88.40) | 7.76 (5.57) | 2.96 (2.06) |
| Insurance issue | 22 | 6.36 (4.71-8.59) | 6.33 (98.50) | 6.31 (4.80) | 2.66 (1.97) |
| Tooth disorder | 20 | 6.45 (4.71-8.84) | 6.43 (91.40) | 6.41 (4.81) | 2.68 (1.96) |
| Injection site irritation | 10 | 8.16 (5.23-12.73) | 8.14 (62.43) | 8.11 (5.40) | 3.02 (1.94) |
| Hypercalcaemia | 12 | 7.43 (4.95-11.14) | 7.41 (66.27) | 7.38 (5.09) | 2.88 (1.92) |
| Spinal stenosis | 8 | 8.86 (5.39-14.56) | 8.84 (55.42) | 8.81 (5.59) | 3.14 (1.91) |
| Lack of injection site rotation | 5 | 11.45 (6.11-21.47) | 11.44 (47.36) | 11.38 (6.40) | 3.51 (1.87) |
| Intracranial pressure increased | 7 | 8.75 (5.15-14.88) | 8.74 (47.76) | 8.70 (5.36) | 3.12 (1.84) |
| Injection site discharge | 7 | 8.37 (4.92-14.22) | 8.35 (45.13) | 8.32 (5.12) | 3.06 (1.80) |
| Self-medication | 5 | 10.21 (5.45-19.13) | 10.20 (41.27) | 10.15 (5.71) | 3.34 (1.78) |
| Attention deficit hyperactivity disorder | 9 | 7.01 (4.39-11.20) | 7.00 (46.15) | 6.98 (4.55) | 2.80 (1.76) |
| Injection site hypersensitivity | 5 | 9.69 (5.17-18.16) | 9.68 (38.73) | 9.64 (5.43) | 3.27 (1.74) |
| Injection site swelling | 47 | 4.37 (3.56-5.37) | 4.33 (120.34) | 4.32 (3.58) | 2.11 (1.72) |
| Incorrect dose administered | 171 | 3.91 (3.51-4.37) | 3.78 (353.34) | 3.78 (3.41) | 1.92 (1.72) |
| SARS-CoV-2 test positive | 21 | 5.07 (3.73-6.90) | 5.05 (68.14) | 5.04 (3.81) | 2.33 (1.72) |
| Laboratory test abnormal | 27 | 4.63 (3.53-6.07) | 4.60 (76.05) | 4.59 (3.59) | 2.20 (1.68) |
| Surgery | 41 | 4.23 (3.39-5.27) | 4.19 (99.73) | 4.19 (3.42) | 2.07 (1.66) |
| Injection site vesicles | 7 | 7.04 (4.14-11.97) | 7.03 (36.10) | 7.01 (4.31) | 2.81 (1.65) |
| Back pain | 114 | 3.71 (3.24-4.23) | 3.62 (217.93) | 3.62 (3.20) | 1.86 (1.62) |
| Energy increased | 6 | 7.27 (4.10-12.90) | 7.26 (32.29) | 7.24 (4.29) | 2.86 (1.61) |
| Spinal operation | 13 | 5.15 (3.49-7.60) | 5.13 (43.18) | 5.12 (3.59) | 2.36 (1.60) |
| Injection site rash | 20 | 4.48 (3.27-6.14) | 4.46 (53.71) | 4.46 (3.34) | 2.16 (1.57) |
| Gastroenteritis viral | 13 | 4.99 (3.38-7.36) | 4.97 (41.19) | 4.96 (3.48) | 2.31 (1.57) |
| Blood calcium decreased | 8 | 5.73 (3.49-9.41) | 5.72 (31.06) | 5.70 (3.62) | 2.51 (1.53) |
| Injection site mass | 28 | 3.84 (2.94-5.00) | 3.81 (58.16) | 3.81 (2.99) | 1.93 (1.48) |
| Arthralgia | 195 | 3.22 (2.91-3.57) | 3.10 (282.61) | 3.10 (2.82) | 1.63 (1.47) |
| Vitamin D deficiency | 7 | 5.52 (3.25-9.38) | 5.51 (25.77) | 5.50 (3.38) | 2.46 (1.45) |
| Prescription drug used without a prescription | 8 | 5.10 (3.10-8.37) | 5.09 (26.21) | 5.08 (3.22) | 2.34 (1.43) |
| Streptococcal infection | 5 | 6.33 (3.38-11.85) | 6.32 (22.32) | 6.30 (3.55) | 2.66 (1.42) |
| Joint stiffness | 14 | 4.17 (2.87-6.07) | 4.16 (33.59) | 4.16 (2.95) | 2.05 (1.41) |
| Product distribution issue | 9 | 4.55 (2.85-7.27) | 4.55 (24.84) | 4.54 (2.96) | 2.18 (1.37) |
| Fear of injection | 7 | 4.94 (2.91-8.39) | 4.93 (21.89) | 4.92 (3.03) | 2.30 (1.35) |
| Foot deformity | 7 | 4.81 (2.83-8.17) | 4.80 (21.02) | 4.79 (2.95) | 2.26 (1.33) |
| Illness | 81 | 2.97 (2.54-3.48) | 2.93 (103.38) | 2.92 (2.53) | 1.55 (1.32) |
| Injection site bruising | 37 | 3.18 (2.52-4.01) | 3.16 (54.64) | 3.15 (2.55) | 1.66 (1.31) |
| Injection site pain | 134 | 2.83 (2.50-3.20) | 2.77 (152.96) | 2.76 (2.47) | 1.47 (1.30) |
| Respiratory syncytial virus infection | 8 | 4.37 (2.66-7.18) | 4.36 (20.70) | 4.36 (2.77) | 2.12 (1.29) |
| Joint dislocation | 6 | 4.83 (2.73-8.57) | 4.83 (18.16) | 4.82 (2.85) | 2.27 (1.28) |
| Musculoskeletal stiffness | 39 | 3.06 (2.44-3.83) | 3.04 (53.40) | 3.03 (2.47) | 1.60 (1.28) |
| Hypophosphataemia | 5 | 5.22 (2.79-9.77) | 5.21 (16.98) | 5.20 (2.93) | 2.38 (1.27) |
| Spinal pain | 8 | 4.23 (2.57-6.94) | 4.22 (19.62) | 4.21 (2.68) | 2.07 (1.26) |
| COVID-19 | 138 | 2.72 (2.41-3.07) | 2.66 (144.29) | 2.65 (2.37) | 1.41 (1.25) |
| Ligament sprain | 6 | 4.30 (2.42-7.62) | 4.29 (15.12) | 4.29 (2.54) | 2.10 (1.18) |
| Therapy interrupted | 47 | 2.60 (2.12-3.19) | 2.58 (45.60) | 2.58 (2.14) | 1.37 (1.11) |
| Nephrolithiasis | 20 | 2.83 (2.07-3.88) | 2.82 (23.53) | 2.82 (2.12) | 1.50 (1.09) |
| Lower limb fracture | 9 | 3.23 (2.02-5.16) | 3.22 (13.80) | 3.22 (2.10) | 1.69 (1.06) |

Abbreviations: PT, preferred term; CI, confidence interval; ROR, reporting odds ratio; PRR, proportional reporting ratio; χ2, chi-squared; IC, information component; IC025, the lower limit of 95 % CI of the IC; EBGM, empirical Bayesian geometric mean; EBGM05, the lower limit of 95 % CI of EBGM.
